# Effects of *BRCA* Germline Mutations on Triple-Negative Breast Cancer Prognosis

**DOI:** 10.1155/2020/8545643

**Published:** 2020-01-27

**Authors:** Katarzyna Pogoda, Anna Niwińska, Elżbieta Sarnowska, Dorota Nowakowska, Agnieszka Jagiełło-Gruszfeld, Janusz Siedlecki, Zbigniew Nowecki

**Affiliations:** ^1^Department of Breast Cancer and Reconstructive Surgery, Maria Sklodowska-Curie Institute–Oncology Center, Warsaw, Poland; ^2^Department of Molecular and Translational Oncology, Maria Sklodowska-Curie Institute–Oncology Center, Warsaw, Poland; ^3^Genetic Counseling Unit, Cancer Prevention Center, Maria Sklodowska-Curie Institute–Oncology Center, Warsaw, Poland

## Abstract

Germline *BRCA1* and *BRCA2* mutations confer an increased lifetime risk for breast cancer and ovarian cancer. Several studies have investigated prognosis among *BRCA1/2* mutation carriers and noncarriers, but the prognostic impact on outcomes of breast cancer patients has not been determined. The aim of this study was to determine the prognosis of TNBC patients with and without *BRCA1/2* germline mutation. Among 502 patients diagnosed with TNBC between 2005 and 2008, 124 patients with a strong family history of breast cancer or ovarian cancer as well as TNBC patients diagnosed under 45 years were referred to the Genetic Counseling Unit for genetic counselling and genetic tests. In 30 (24%) of them, the *BRCA1/2* mutation was detected (the most common 5382insC in 18 (60%) patients). The median follow-up of the entire group was 60 months. *BRCA1/2* mutation carriers were statistically significantly younger at TNBC diagnosis compared with nonmutation patients (41 vs 47 years, respectively). Patients with the *BRCA1/2* mutation had smaller tumors (stage I: 47% vs 24.5% in noncarriers), but there was no significant difference in the regional nodal status (58.5–63% with cN0). Contralateral breast cancer developed in 26.5% of *BRCA1/2* mutation carriers and in 14% of noncarriers. Other primary cancers were also slightly more common in *BRCA1/2* mutation carriers (16.5% vs 9.5%). The performed analysis did not show any significant differences between the groups in recurrence-free survival (*p*=0.312). There was no significant difference between patients with or without *BRCA1/2* mutation as regards overall survival (*p*=0.649) and the risk of TNBC death (*p*=0.333). The survival from detection of metastases was similar in two groups (*p*=0.865). Our study demonstrated that the *BRCA1* mutation does not affect TNBC patients' outcomes.

## 1. Introduction

*BRCA1* and *BRCA2* are tumor suppressor genes involved in DNA damage repair, cell cycle control, gene transcription regulation, and apoptosis. The common germline mutations of the *BRCA1* gene are 5382insC, 185delAG, 3819del5, and 4153delA and of *BRCA2* are 4075delGT and 580del4 [1]. In the western population, about 5% of the breast cancer patients may carry heritable cancer susceptibility gene mutations, with *BRCA1* being the most common mutation [2]. The mutation rate can be higher in Ashkenazi Jews [3, 4]. Interestingly, *BRCA1/2* mutation rates in Asians are lower than those in whites [5].

### 1.1. Prevalence of Breast/Ovarian Cancer

Germline *BRCA1* and *BRCA2* mutations confer an increased lifetime risk for breast cancer and ovarian cancer. Women with *BRCA1/2* germline mutations have a higher incidence of breast cancer than those without these genetic abnormalities. The cumulative incidence of breast cancer by age 70–80 years in female mutation carriers is 71.4–87% for the *BRCA1* mutation and 77–88% for the *BRCA2* mutation [6–8]. The ovarian cancer risk is 59–65% for the *BRCA1* mutation and 34.5–37% for the *BRCA2* mutation [6, 8]. The high lifetime risk of breast and ovarian cancers in *BRCA1/2* carriers is crucial for counselling, intensive breast and ovarian screening (annual MRI commenced from the age of 25 with the additional annual mammography from the age of 30, 6-monthly ovarian cancer screening with transvaginal ultrasound, and Ca125 serum measure started at the age of 30), and risk-reducing surgery (bilateral salpingo-oophorectomy and bilateral risk-reducing mastectomy including skin-sparing and nipple-sparing mastectomy) [9, 10].

Compared to *BRCA2* carriers and noncarriers, *BRCA1*-associated breast cancers are often high-grade and poorly differentiated infiltrating ductal carcinoma and are more often triple-negative with higher expressions of cytokeratin 5/6, cyclin E, and p53. Patients with *BRCA1*-associated breast cancers are younger than those with the *BRCA2* mutation and those without mutation [11, 12].

### 1.2. Prognosis

Several studies have investigated prognosis among *BRCA1/2* mutation carriers and noncarriers, but the prognostic impact on outcomes of breast cancer patients has not been definitely determined. It is controversial whether *BRCA1/2* mutations in breast cancer are associated with poor prognosis. Some studies revealed that *BRCA1/2* mutation carriers with breast cancer had worse overall survival (OS) than noncarriers [13–15], others showed no difference [16–20], and some studies indicated that *BRCA1/2* mutation carriers had better survival than noncarriers [21–23]. Differences could be partly the result of the analysis of different ethnic populations (Ashkenazi Jewish population [24], central-eastern population [15], western population [19], or Asian population [20, 25]), small study group with mutations, variations in mutation assay techniques, mutation types, cancer treatment modalities, or length of follow-up.

Among all biological subtypes of breast cancer, triple-negative breast cancer (TNBC) is more likely to harbor a germline *BRCA1/2* mutation, with reported prevalence rates varying from about 10% to 20% [20, 22, 26, 27]. The effect of the *BRCA1/2* mutation on the prognosis in TNBC patients has not been well examined, with divergent findings reported in the previous studies [18, 20, 22, 28–30].

## 2. Aim

The aim of this study was to determine the prognosis of TNBC patients with and without *BRCA1/2* germline mutation.

## 3. Materials and Methods

Five hundred two consecutive TNBC patients treated at the Department of Breast Cancer and Reconstructive Surgery, Maria Skłodowska-Curie Institute–Cancer Center (MSCI), Warsaw, Poland, between 2005 and 2008, were selected and analyzed to assess risk factors of recurrence, recurrence-free survival (RFS), and OS. Among them, 124 patients with a strong family history of breast cancer or ovarian cancer as well as TNBC patients diagnosed under 45 years were referred to the Genetic Counseling Unit of Cancer Prevention Department in MSCI, Warsaw, for genetic counselling and genetic tests. The patients were tested for the following *BRCA1/2* mutations: *BRCA1* gene: c.5266dupC (5382insC), c.181T>G (C61G, 300T>G), c.3700_3704delGTAAA (3819del5), c.68_69delAG (185delAG), c.676delT (p.Cys226Valfs), c.1687C>T (p.Gln563Ter), c.3756_3759delGTCT (3875del4), c.4035delA (4153delA), c.5251C>T (5370C>T), and c.5345G>A (p.Trp1782X) and *BRCA2* gene: c.658_659del GT (p.Val220fs), c.5946delT (6174delT), c.9371A>T (p.Asn3124Ile), and c.5744C>T (C5972T). Characteristics of the whole group of 502 TNBC patients and 124 patients in whom genetic tests were performed are presented in Tables 1 and 2. The Ki-67 expression and vimentin expression were conducted additionally due to the fact that, in the analyzed period of time, these markers were not assessed as standard practice (vimentin still remains as an experimental biomarker, expressed more often in mesenchymal tumors). The decisions on therapy were made regardless of the *BRCA1/2* mutation status.

### 3.1. Statistical Analysis

Univariate analysis was performed in order to compare patient and tumor characteristics (age at diagnosis, clinical stage, HER2 expression, histological grade G, Ki-67 expression, and vimentin expression) as well as therapy (type of surgery, radiotherapy, and (neo)adjuvant chemotherapy) depending on the *BRCA1/2* mutation status. R Development Core Team (R 3.1.3., 2009) software was used for these analyses.

The following definitions of events were used:

- RFS—time from TNBC diagnosis to recurrence
- OS—time from TNBC diagnosis to death from any cause
- Breast cancer-specific survival (BCSS)—time from TNBC diagnosis to death from breast cancer
- Survival from dissemination—time from recurrence to death from any cause

Then, RFS, OS, and survival from dissemination of the disease in both groups were assessed. Additionally, risk of breast cancer death using the competing risk method was evaluated. Finally, the *BRCA1/2* mutation was assessed as one of the seven prognostic factors for recurrence and survival in multivariate analysis using the multistep Cox model. The other prognostic factors in the Cox model were age at diagnosis, TNM stage (I, II, or III), Ki-67 expression, vimentin expression, histological grade G (G1, G2, or G3), and histological type (no special type—NST or others).

## 4. Results

Finally, 124 (25%) out of 502 TNBC patients had undergone genetic counselling with *BRCA1/2* mutation tests and were included for further analysis. In 30 (24%) of them, the *BRCA1/2* mutation was detected. Only in one case, the mutation of the *BRCA2* gene was found, and for the *BRCA1* gene, 29 mutated cases were detected. The following *BRCA1* mutations were found: c.5266dupC (5382insC) in 18 patients, c.181T>G (C61G, 300T>G) in 5 patients, c.3700_3704delGTAAA (3819del5) in 2 patients, and c.5251C>T (5370C>T), c.5345G>A (p.Trp1782X), c.3756_3759delGTCT (3875del4), and c.68_69delAG (185delAG) in 1 patient each, respectively. One patient harbored *BRCA2* gene mutation c.5744C>T (C5972T). The comparison between *BRCA1/2* mutation carriers and noncarriers is presented in Table 2. The median follow-up of the entire group was 60 months. *BRCA1/2* mutation carriers were statistically significantly younger at TNBC diagnosis compared with nonmutation patients (41 vs 47 years, respectively). Patients with the *BRCA1/2* mutation had smaller tumors (stage I: 47% vs 24.5% in noncarriers), but there was no significant difference in the regional nodal status (58.5–63% with cN0). The most common histological type was NST in both groups with a similar rate of medullar cancer (3.5–5.5%). Noncarriers had more often G3 tumors. Contralateral breast cancer developed in 26.5% of *BRCA1/2* mutation carriers and in 14% of noncarriers. In both groups, almost half contralateral breast cancers developed before TNBC diagnosis. Other primary cancers were also slightly more common in *BRCA1/2* mutation carriers (16.5% vs 9.5%). Almost all cases occurred after TNBC diagnosis in both groups (only 2 cases of lymphoma and one ovarian cancer developed before TNBC). The summary of these results is presented in Table 2.

In 72 patients (58% of all TNBC), the primary operation was performed. In other 47 (38%) patients, surgery was carried out after neoadjuvant chemotherapy. Breast-conserving surgery was more common in *BRCA1/2* mutation carriers (41.5% vs 33.5%). Adjuvant chemotherapy was performed in 87 patients (90% after primary surgery). Overall, (neo)adjuvant chemotherapy was performed in a similar percentage of patients with or without *BRCA1/2* mutation. The summary of patient therapy is presented in Table 3.

**Figure 1:**
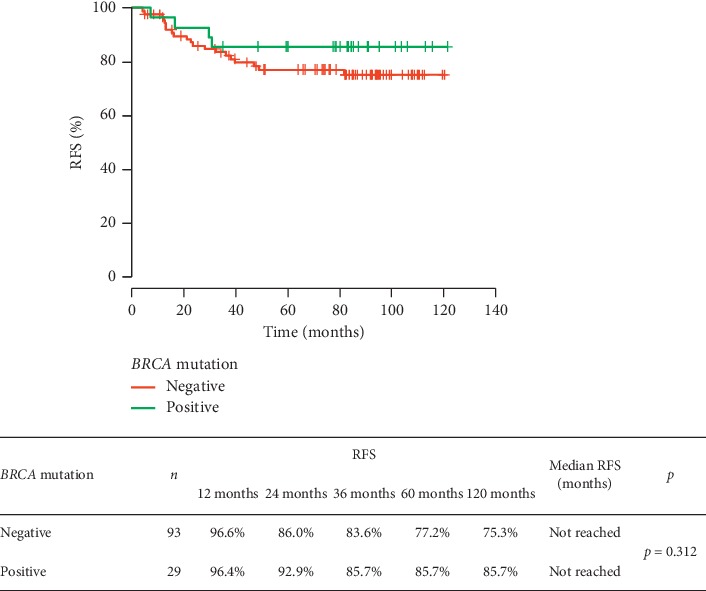
Risk of recurrence in TNBC patients depending on the *BRCA* mutation status.

**Figure 2:**
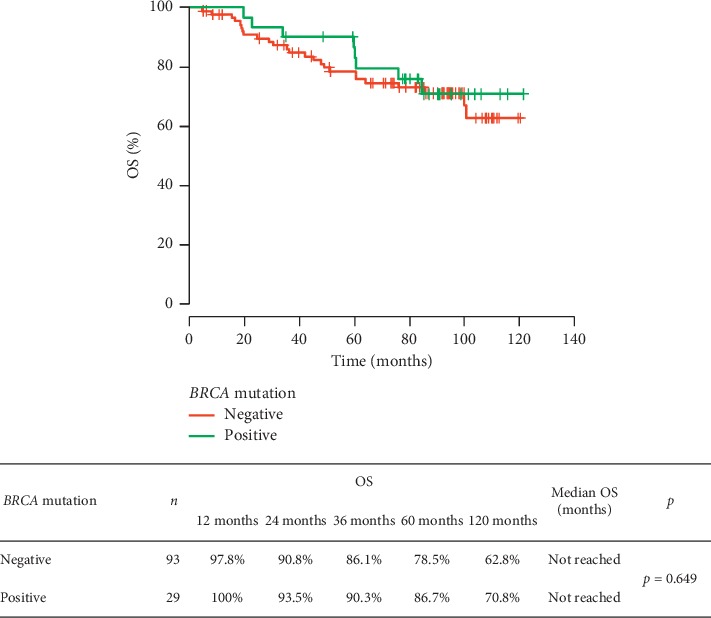
Risk of death in TNBC patients depending on the *BRCA* mutation status.

**Figure 3:**
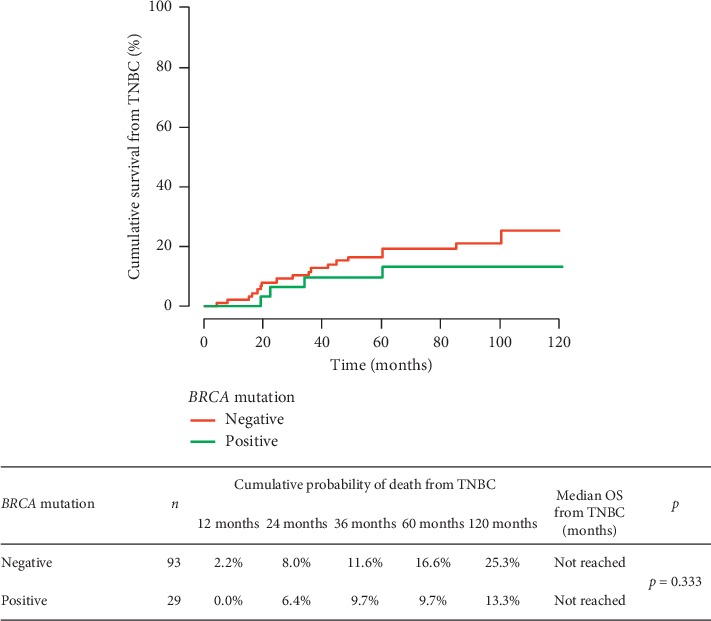
Relationship between the presence of the *BRCA1/2* mutation and the risk of death due to TNBC.

We compared RFS, OS, risk of breast cancer death, and survival from distant metastases in *BRCA1/2* carriers and noncarriers. The performed analysis did not show any significant differences between the groups in RFS (*p*=0.312), also after taking into account the clinical stage of TNBC (in patients in the following stages: I: *p*=1.0, II: *p*=0.454, and III: *p*=0.197) or (neo)adjuvant chemotherapy (*p* > 0.05). The risk of the recurrence depending on the *BRCA1/2* mutation status is shown in Figure 1. There was no significant difference between patients with or without *BRCA1/2* mutation regarding overall survival (*p*=0.649). The *BRCA1/2* mutation was not a prognostic factor of patient survival. The results are presented in Figure 2. The risk of TNBC death did not differ significantly in both groups (Figure 3).

**Figure 4:**
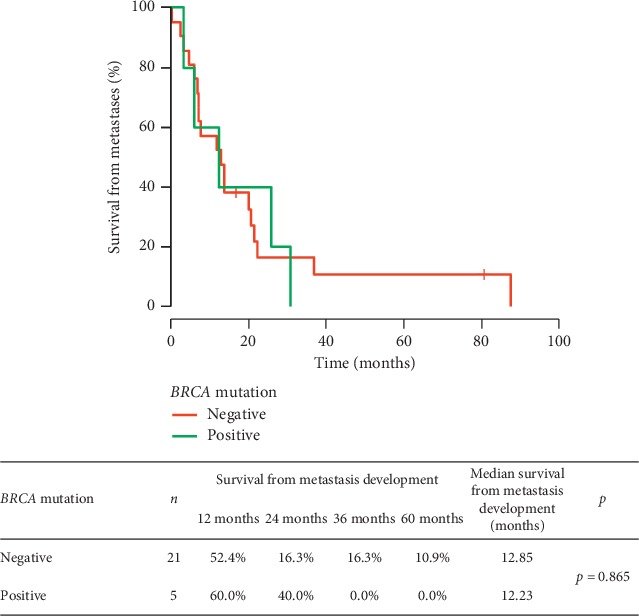
Survival time counted from relapse depending on the *BRCA1/2* mutation status.

In 13% (4/30) of *BRCA1/2* mutation patients and in 21% (20/94) of noncarriers, the recurrence of the disease was detected. In both groups, there was one patient with primary metastatic TNBC. There was no significant difference in survival from detection of metastases between these two groups (*p*=0.865). The results are presented in Figure 4.

Among seven variables taken in multivariate analysis, TNM stage was the only factor significantly influencing recurrence and death. There was no correlation between RFS or OS and other analyzed risk factors, including the *BRCA1/2* germline mutation. The results are shown in Tables 4 and 5.

## 5. Discussion

Our study showed that the outcome of TNBC patients did not differ depending on the *BRCA* mutation status. We aimed to clarify the prognostic value of *BRCA1/2* mutations on breast cancer-specific outcomes after conventional treatment. In our study, RFS, OS, and risk of death from TNBC were similar between patients with breast cancer and *BRCA1* germline mutation and noncarriers. Because of the fact that among our patients with *BRCA1/2* mutations only one had *BRCA2* mutation, the results and discussion concern about patients with breast cancer and *BRCA1* mutation.

### 5.1. All Biological Types of Breast Cancer

The meta-analysis of 11 studies performed by Lee et al. revealed that patients with breast cancer and *BRCA1* mutation had worse OS compared to noncarriers (HR = 1.92). The *BRCA2* mutation did not affect survival in patients with breast cancer (HR = 1.30) [31].

In meta-analysis by Zhong et al. [32], based on 13 studies with 10 016 women with breast cancer, concerning breast cancer survival, the *BRCA1* mutation carriers had worse OS than noncarriers (HR = 1.5, *p*=0.009) but were not significantly different from noncarriers in terms of progression-free survival (HR = 1.35, *p*=0.09).

In other meta-analysis performed by Zhu et al. [3], based on 34 studies, event-free survival (EFS), OS, and BCSS were compared in three groups of breast cancer patients: *BRCA1* carriers, *BRCA2* carriers, and *BRCA1/2* noncarriers. In patients with *BRCA1* and *BRCA2* mutations, OS was worse than that in patients without mutation (*p* < 0.001 and *p*=0.034, respectively) but did not translate into poor BCSS (*p*=0.448 and *p*=0.401, respectively) or EFS (*p*=0.438 and *p*=0.558, respectively) [3]. The *BRCA1* mutation was significantly associated with worse OS in studies conducted in Europe (*p* < 0.001) and studies assessing patients diagnosed before 1995 (*p* < 0.007).

The POSH prospective cohort study analyzed patients with young-onset breast cancer (≤40 years) regarding the *BRCA1/2* mutation status [33]. Recently published results indicated no significant difference in OS or distant disease-free survival between patients carrying *BRCA1/2* mutations and patients without those mutations after a diagnosis of breast cancer.

A study by Wang et al. performed on the Chinese cohort revealed that patients with *BRCA1/2* mutations had worse survival outcomes than noncarriers [25]. *BRCA1/2* mutation carriers were more likely to have lymph node involvement at initial diagnosis than noncarriers [25]. In our study, we did not observe these kinds of relations.

### 5.2. Triple-Negative Breast Cancer

Studies that have evaluated the prognostic role of the *BRCA1/2* mutation in patients with TNBC have shown inconclusive results, but the newest and larger ones are in line with our study.

In the study performed by Yadav et al. [34], 266 TNBC patients had undergone *BRCA1/2 mutation* tests. In 27% of them, *BRCA1/2* mutations were detected. No statistically significant difference was found in locoregional recurrence, distant recurrence, RFS, and OS between the breast cancer patients with and without *BRCA1/2* mutations. 5-year OS for *BRCA1/2*-positive and *BRCA1/2*-negative breast cancer patients was 83% and 90% and 5-year RFS was 83% and 80%, respectively. The differences were not statistically significant [34].

In the study by Gonzales-Angulo et al. [22], based on 77 TNBC patients, RFS was better for patients with the *BRCA1/2* mutation and OS was similar between carriers and noncarriers.

In another study, Maksimenko et al. [30] compared the outcomes of 78 TNBC patients without *BRCA1* mutation with those of 38 TNBC patients with the *BRCA1* mutation. The BCSS and distant recurrence were significantly lower in the *BRCA1*-positive patients. In 4 other larger studies, there was no difference found in recurrence and survival between TNBC carriers and noncarriers of *BRCA1/2* mutations [18, 20, 28, 29]. A meta-analysis of 11 papers performed by Xie et al. also revealed that RFS and OS in TNBC patients with and without *BRCA1/2* mutations did not differ [20].

Baretta et al. [24] performed a meta-analysis concerning the relation between *BRCA1/2* mutation and prognosis of breast cancer based on 105 220 breast cancer patients including 3588 (3.4%) *BRCA1/2* mutation carriers. OS, BCSS, RFS, and distant metastasis-free survival (DMFS) were estimated. The authors found that *BRCA1* mutation carriers had a 30% higher risk of dying than *BRCA1*-negative/sporadic cases (OS), but they did not find association between *BRCA1* and the risk of death from breast cancer (BCSS). Contrary to patients with all subtypes of breast cancer, 1748 patients with TNBC and *BRCA1/2* mutations had better OS than *BRCA1/2*-negative ones (HR = 0.49) [24]. The risk of recurrence in TNBC was not statistically different between *BRCA1/2* carriers and *BRCA1/2* noncarriers (*p*=0.82). BCSS and DMFS of *BRCA1* mutation carriers did not differ from those of *BRCA1*-negative TNBC patients (*p*=0.76 and *p*=0.65, respectively) [24].

In the present study, all investigated TNBC cases were diagnosed and treated in one breast cancer department. The used methods did not differ depending on the *BRCA1/2* mutation status, and patients had a long time of follow-up (up to 10 years). Nowadays, new drugs such as poly(adenosine diphosphate-ribose) polymerase (PARP) inhibitors (olaparib and talazoparib) are dedicated to metastatic *BRCA1/2*-positive TNBC as well as immunotherapy for PDL-1-positive metastatic TNBC [35–37]. These drugs can influence the survival of *BRCA1/2* carriers with TNBC in the future. In the analyzed cohort with metastatic disease, the survival did not depend on the *BRCA1/2* mutation status. In contrast, Larson et al. showed that *BRCA* carriers with metastatic TNBC had clinically significant improved OS at 3 years compared to patients without *BRCA* mutations (3-year OS of 63% vs 28%). In that study also, no patients received treatment with the PARP inhibitor [38].

## 6. Limitations of the Study

The retrospective nature of the study and a small number of recurrences or deaths in patients who had undergone genetic tests are two main limitations of this study.

Out of 502 consecutive TNBC patients referred to MSCI between the years 2005 and 2008, only 124 (25%) patients underwent genetic tests for the *BRCA1/2* mutation. From them, the *BRCA1/2* mutation was found only in 30 cases, which gives 6% (30/502) *BRCA1/2* carriers among 502 TNBC patients. According to the current NCCN guideline and ESMO recommendations, 65% of all TNBC patients from our analysis met the genetic test criteria solely by their age at diagnosis of TNBC (up to 60 years); therefore, the tests should be performed [10, 39]. This number might be even higher considering other criteria such as a strong family history of breast/ovarian cancer. In the years 2005–2008, genetic tests were offered at our institution only for patients with a strong family history of breast/ovarian cancer and for those under 45 years at the initial diagnosis of breast cancer.

## 7. Conclusion

Our study demonstrated that the *BRCA1* mutation does not affect RFS and OS in patients diagnosed with TNBC. The outcome of breast cancer in *BRCA1* carriers and noncarriers was comparable. The *BRCA1* germline mutation did not influence the prognosis of the TNBC patients.

## Figures and Tables

**Table 1:** Characteristics of 502 TNBC patients.

Factor		Rate (%)
Number of patients	502	100

*Age at diagnosis (years)*		
Median	55	
Mean	**56**	
Range	24–98	

*Clinical staging (cTNM)*		
I	97	19.5
II	246	49
III	132	26
IV	27	5.5

*Initial clinical tumor staging*		
cT0	7	1
cT1	111	22
cT2	248	49.5
cT3	58	12
cT4	76	15
No available data	2	0.5

*Initial clinical node staging*		
cN0	243	48
cN1	180	36
cN2	58	11.5
cN3	19	4
No available data	2	0.5

*HER2 expression*		
0 or 1+	431	86
2+, FISH negative	71	14

*Histological type*		
NST	416	83
Lobular	25	5
Medullar	11	2
Apocrine	11	2
Metaplastic	20	4
Others	20	4

*G*		
1	21	4
2	165	33
3	310	62
No available data	6	1

*Ki-67 expression*		
<14%	140	28
14–30%	183	36.5
>30%	133	26.5
No available data	46	9

*Vimentin expression assessed*		
Yes	443	88
No	59	12

*Vimentin*		
Positive	71/443	16
Negative	372/443	84

Contralateral breast cancer	41	8
Other primary cancer (other than contralateral breast cancer)	45	9

FISH: fluorescence in situ hybridization.

**Table 2:** Characteristics of 124 TNBC patients assessed for *BRCA1/2* mutations.

Factor	Patients tested for *BRCA* mutations				*p* value (*BRCA*-positive vs *BRCA*-negative)
	*BRCA* noncarriers	Rate (%)	*BRCA* carriers	Rate (%)	
Number of patients	94	100	30	100	

*Age at diagnosis (years)*					
Median	49		40		0.0115
Mean	47.5		41.4		
Range	25–67		24–76		

*Clinical staging (cTNM)*					
I	23	24.5	14	47	0.0006
II	51	54	13	43	
III	19	20	2	7	
IV	1	<0.5	1	3	

*Initial clinical tumor staging*					
cT0	0	0	0	0	0.0004
cT1	28	30	16	53	
cT2	56	59.5	9	30	
cT3	4	4	2	7	
cT4	6	6.5	3	10	
No available data	0	0	0	0	

*Initial clinical node staging*					
cN0	55	58.5	19	63	0.1063
cN1	27	28.5	10	33	
cN2	9	9.5	1	4	
cN3	3	3.5	0	0	
No available data	0	0	0	0	

*HER2 expression*					
0 or 1+	79	84	29	97	0.0091
2+, FISH negative	15	16	1	3	

*Histological type*					
NST	80		21	70	0.0023
Lobular	5	85	1	3.5	
Medullar	5	5.5	1	3.5	
Apocrine	2	5.5	1	3.5	
Metaplastic	2	2	2	6	
Others	0	2	4	13.5	

*G*					
1	0	0	2	6.5	0.0065
2	29	30	12	40	
3	64	68	16	53.5	
No available data	1	2	0	0	

*Ki-67 expression*					
<14%	26	27.5	5	16.5	0.0761
14–30%	29	31	10	33.5	
>30%	28	30	13	43.5	
No available data	11	11.5	2	6.5	

*Vimentin expression assessed*					
Yes	82	87	26	86.5	0.8361
No	12	13	4	13.5	

*Vimentin*					
Positive	14	15	8	26.5	0.0372
Negative	68	85	18	73.5	

Contralateral breast cancer	13	14	8	26.5	0.0228
Other primary cancer (other than contralateral breast cancer)	9	9.5	5	16.5	0.1475

FISH: fluorescence in situ hybridization.

**Table 3:** Therapy of 124 TNBC patients assessed for *BRCA1/2* mutations.

Type of therapy	Patients tested for *BRCA* mutations				*p* value (*BRCA*-positive vs *BRCA*-negative)
	*BRCA* noncarriers	Rate (%)	*BRCA* carriers	Rate (%)	
Number of patients	94	100	30	100	

*Surgery*					
Yes	90	96	29	97	0.7004
No	4	4	1	3	

*Type of surgery*					
Mastectomy	60/90	66.5	17/29	58.5	0.2438
Breast-conserving surgery	30/90	33.5	12/29	41.5	

*Radiotherapy*					
Yes	55	58.5	17	56.5	0.7751
No	39	41.5	13	43.5	

*Radiotherapy*					
After mastectomy	27/55	49	5/17	29.5	0.0044
After breast-conserving surgery	28/55	51	12/17	70.5	

*Neoadjuvant chemotherapy*					
Yes	20	21.5	4	13.5	0.0940
No	74	78.5	26	86.5	

*Regimens in neoadjuvant chemotherapy*					
AT⟶CMF	5/20	25	1/4	25	<0.0001
Anthracycline + taxane	9/20	45	2/4	50	
Anthracycline	5/20	5	1/4	25	
Others	1/20	25	0	0	

*Adjuvant chemotherapy*					
Yes	64	68	23	76.5	0.1541
No	30	32	7	23.5	

*Regimens in adjuvant chemotherapy*					
Anthracycline (AC)	41/64	64	12/23	52	0.0574
FEC/FAC	11/64	17.5	4/23	17.5	
Anthracycline + taxane	8/64	12.5	5/23	21.5	
CMF	2/64	3	0	0	
Taxane	2/64	3	2/23	9	

**Table 4:** Multivariate analysis: final model for RFS.

Factor	HR	95% CI		*p*
Clinical stage: I or II	Reference			

Clinical stage: III	43.26	2.13	880.64	0.014

**Table 5:** Multivariate analysis: final model for OS.

Factor	HR	95% CI		*p*
Clinical stage: I	Reference			

Clinical stage: II	2.359	1.385	4.016	0.002

Clinical stage: III	8.353	4.918	14.188	<0.001

## Data Availability

The data used to support the findings of this study are available from the corresponding author upon reasonable request.
